# Longitudinal assessment and stability of long non-coding RNA gene expression profiles measured in human peripheral whole blood collected into PAXgene blood RNA tubes

**DOI:** 10.1186/s13104-020-05360-3

**Published:** 2020-11-12

**Authors:** Lukasz S. Wylezinski, Guzel I. Shaginurova, Charles F. Spurlock III

**Affiliations:** 1 IQuity, Inc, 111, 10th Avenue South, Suite 100, Nashville, TN 37203 USA; 2 Decode Health, Inc, 209 10th Avenue South, Suite 404, Nashville, TN USA; 3 Department of Medicine, Vanderbilt University School of Medicine, 1161 21st Avenue South, Medical Center North T3113, Nashville, TN USA

**Keywords:** Long non-coding RNA, Multiple sclerosis, Messenger RNA, PAXgene blood RNA tube, Quantitative real time PCR, Storage

## Abstract

**Objective:**

Long non-coding RNAs (lncRNAs) are emerging as novel biomarkers for a variety of chronic conditions including autoimmune disease. PAXgene Blood RNA tubes are routinely used in clinical research and molecular diagnostic development to capture RNA profiles in peripheral whole blood. While the stability of mRNA expression profiles captured using PAXgene tubes has been documented previously, no previous work has determined the stability of lncRNA expression profiles observed in PAXgene tubes stored at − 80 °C. Here we sought to determine the effects on lncRNA expression profiles following − 80 °C storage of total RNA templates, cDNA synthesized using fresh or frozen total RNA template, and the impact of freeze–thaw cycles on both total RNA and cDNA obtained from PAXgene tubes.

**Results:**

We find that storage of whole blood in PAXgene tubes, total RNA and cDNA for up to 1 year at − 80 °C or up to ten total RNA or cDNA freeze–thaw cycles do not significantly alter lncRNA expression profiles compared to baseline. As monthly expression profiles were determined, some month to month lncRNA expression variability was observed. However, all monthly observations fell within the 95% confidence interval calculated at baseline.

## Introduction

Long non-coding RNAs (lncRNAs) play pivotal roles in gene regulation, protein synthesis, sex chromosome compensation and telomere maintenance [1–5]. Work over the past decade has also implicated specific lncRNAs in a variety of pathological processes and human diseases including cancer, autoimmune disease and neurodegenerative disorders like Parkinson’s and Alzheimer’s [6–13]. Apart from mechanistic studies ascribing biological outcomes to over- or under-expression of specific lncRNAs, lncRNAs that exhibit expression differences are often proposed as candidate biomarkers that could be measured in novel RNA-based assays to aid in the diagnosis of disease or to monitor disease progression [14–17]. Our own cross-sectional studies in human disease have included extensive RNA sequencing and quantitative real time PCR (qRT-PCR) studies to measure a variety of RNA species including protein-coding and non-coding genes, with a particular focus on lncRNAs. We identified a series of mRNAs, annotated and novel lncRNA genes that are differentially expressed among relapsing–remitting multiple sclerosis patients, healthy control subjects, and disease controls [16, 18–20]. These expression differences were measured in peripheral whole blood samples collected into PAXgene Blood RNA tubes. Previous studies have demonstrated the relative stability of messenger RNA (mRNA) expression after PAXgene tube storage at − ‍70 °C [21] for up to 11 years [22]. To our knowledge, no previous research has determined whether extended storage of whole blood collected into PAXgene tubes could alter lncRNA expression. Examining whether differences in lncRNA expression measurements are produced from cDNA synthesized using freshly isolated total RNA template or total RNA template stored at − 80 °C for periods of up to 1 year is also incompletely understood. Additionally, no previous work has documented the impact of multiple freeze–thaw cycles of total RNA template or synthesized cDNA on lncRNA qRT-PCR studies. Given the potential utility of lncRNAs in peripheral whole blood to serve as biomarkers for disease, we conducted a 1 year stability study of lncRNA expression profiles using peripheral whole blood collected and stored in PAXgene tubes.

## Main text

### Methods

#### Blood collection, RNA isolation, cDNA synthesis, and nucleic acid assessment

RNA was isolated from peripheral whole blood collected into PAXgene Blood RNA tubes (PreAnalytiX) according to the manufacturer’s supplied protocol using an automated QIAcube system (Qiagen). RNA samples were further purified, concentrated, and eluted in RNase-free water using the RNEasy MinElute Cleanup Kit (Cat. No. 74204, Qiagen). cDNA synthesis was performed using the Superscript III First-Strand Synthesis System with oligo-dT as primer (Cat. No. 18080051, Invitrogen). cDNA samples were purified and eluted in elution buffer (10 mM TrisCl, pH = 8.5) using the QiaQuick PCR Purification Kit (Cat. No. 28106, Qiagen) prior to qRT-PCR measurements. Total RNA and cDNA were assessed for quantity and purity using a Nanodrop 1000 spectrophotometer (ThermoFisher) prior to storage at − 80 °C in 1.5 mL Eppendorf tubes. RNA integrity number (RIN) values were obtained using the RNA 6000 Nano Total RNA Kit and a Bioanalyzer 2100 instrument (Cat. No. 5067-1511, Agilent Technologies, Inc.). All above-mentioned steps were performed according to the manufacturer’s protocols. Freeze–thaw studies were performed by thawing RNA samples and incubating the sample for 10 min at room temperature (21 °C) and re-freezing the sample at − 80 °C for 30 min.

#### qRT-PCR measurements

Custom DNA oligos were synthesized (Integrated DNA Technologies, Inc.) that target specific lncRNA and mRNA genomic loci (Additional file 1: Table S1). Quality control steps were performed prior to qRT-PCR studies. Single PCR products were confirmed by agarose gel electrophoresis and amplified PCR products were verified by Sanger Sequencing (Genewiz) (data not shown). qRT-PCR measurements were performed using a QuantStudio 12 K Flex PCR system (Life Technologies). cDNA samples were loaded into 384-well plates in a 10µL final volume with PowerUp SYBR Green Master Mix (Applied Biosystems) and a final primer concentration of 0.5 µM. 384-well plates were pre-loaded with primers using automated liquid handler VIAFLO96/384 (Integra Biosciences Corp.). Expression values were normalized to *GAPDH* using the $\mathrm{\Delta \Delta }$Ct method as previously described [23, 24].

#### Statistical analysis

Statistical analysis was performed using GraphPad Prism 8.3.1 and denoted within each figure legend.

## Results

### Evaluation of lncRNA expression following storage of PAXgene tubes, RNA and cDNA for up to one year

**Fig.1:**
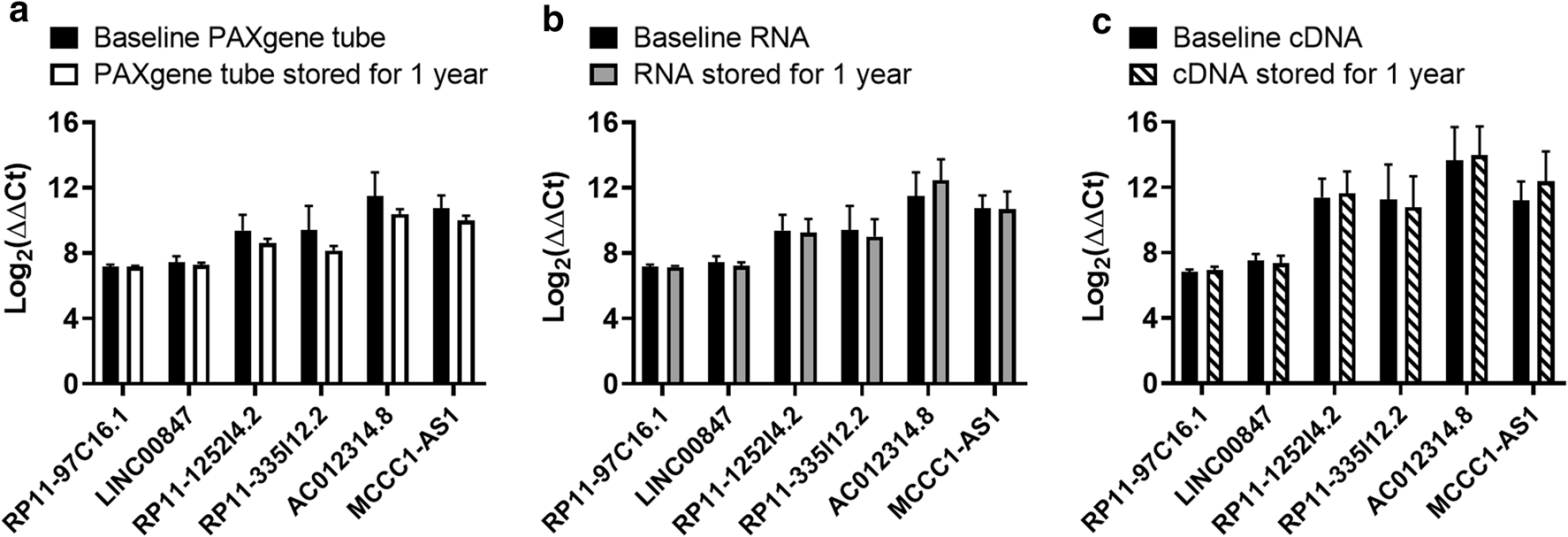
Annotated lncRNA expression values do not differ significantly after storage for 1 year at − 80 °C. lncRNA expression levels were measured using **a** total RNA immediately isolated from PAXgene tubes at baseline or RNA isolated after PAXgene tubes were stored at − 80 °C for one year, **b** total RNA isolated at baseline compared to the same RNA stored at − 80 °C for one year, or **c** cDNA immediately prepared using baseline total RNA or the same cDNA sample stored for one year at − 80 °C. Student’s t-test with Welch’s correction was used to determine statistical significance. Bars represent mean Log_2_(ΔΔCt) with SD for n = 5 PAXgene tubes with pooled blood, RNA samples or cDNA samples in each experimental group

To examine the effects of various storage conditions on lncRNA expression, we initiated our study utilizing a panel of annotated lncRNAs from previous RNA sequencing studies that were conducted across multiple human autoimmune diseases (Additional file 1: Table S1 and Additional file 2: Fig.S1). These studies identified annotated lncRNAs that were differentially expressed among multiple sclerosis and healthy control subjects and were further validated by qRT-PCR measurements [18]. Six of these annotated lncRNAs were selected to assess the stability of RNA transcripts across various storage conditions: PAXgene tube, purified total RNA, and synthesized cDNA. A schematic of experimental steps is presented in Additional file 3: Fig.S2. Peripheral whole blood from five healthy donors was collected into twelve PAXgene tubes per individual. Samples were pooled to create a homogenous mixture and then aliquoted equally into sixty tubes. Total RNA was immediately isolated from five PAXgene tubes, cDNA was synthesized and expression levels of the six annotated lncRNAs measured to establish a baseline expression profile. The additional tubes were frozen at − 80 °C for use at future timepoints. The remaining eleven experimental groups of five PAXgene tubes were stored and isolated each month over the course of 1 year. Expression of annotated lncRNAs isolated at baseline were compared to expression of lncRNAs from PAXgene tubes where RNA was isolated and cDNA was synthesized 1 year later. Whether total RNA was isolated from PAXgene tubes immediately or whole blood lysates were stored with total RNA purified 1 year later, no statistically significant differences in lncRNA expression were found (Fig. 1a). Stability of purified total RNA (Fig. 1b) and cDNA (Fig. 1c) was then compared at baseline and after storage for 1 year. Similar to what was observed in RNA isolated immediately or after 1 year from each PAXgene tube, no significant difference was observed across the six annotated lncRNAs we measured following storage of total RNA or cDNA.

During the course of the study, consecutive, monthly assessments were also performed whereby five PAXgene tubes were pulled from storage and expression levels of each lncRNA was analyzed for a total of twelve series of qRT-PCR measurements (Additional file 4: Fig.S3). Across each monthly experiment, the 95% CI was calculated and compared to baseline lncRNA expression values after normalization to *GAPDH*. No significant differences were observed for *RP11-97C16.1* or *LINC00847*. However, significant differences in the 95% CI were observed at month 10 for *RP11-125,214.2, AC012314.8,* and *RP11-335I12.2*. Additionally, a significant difference in the 95% CI was observed in months 2, 4, 6, and 7 for *MCCC1-AS1*. On a monthly basis we saw a stochastic pattern of lncRNA expression oscillating around the baseline level established. lncRNAs with lower expression levels, in particular *MCCC1-AS1*, with a mean Ct value of 29.73, exhibited multiple months of statistically significant differences in expression. When baseline values were compared to each monthly observation, none of the lncRNAs fell outside of the 95% baseline CI established at the start of the study. Furthermore, in five of the six lncRNAs, all monthly 95% CI overlapped. In *MCCC1-AS1*, however, the 95% CI at months 2, 4, 6, and 7 did not overlap when these individual months were compared indicating a pattern of greater variability in this particular gene target. This is consistent with other studies that have noted greater experimental variation among genes with higher Ct values [25]. Furthermore, it has been shown that lncRNAs display cell type specificity as well as a wide range of intrinsic stability [26, 27]. Inherently, more stable lncRNAs will be better equipped to endure storage conditions. These differences become more pronounced when measuring lncRNAs expressed at low levels. We believe this phenomenon is observed in the variability of *MCCC1-AS1* measurements as compared to *AC012314.8*.

Quality metrics for all RNA samples processed during the study were also assessed (Additional file 5: Table S2). Each PAXgene tube containing pooled blood from five donors yielded, on average, between 2.72 and 7.98 µg of purified total RNA with no discernible correlation between length of storage at − 80 °C and total amount of RNA isolated (Additional file 6: Fig.S4). During the course of our study, the coefficient of variation calculated for RNA yield ranged from 4.41% to 19.37% (Additional file 6: Fig.S4d). This falls within the range previously reported by the manufacturer and cleared by the Federal Drug Administration in its approval of PAXgene tubes as medical devices [28].

### Evaluation of RNA integrity number, lncRNA and mRNA expression following freeze–thaw cycles

**Fig. 2:**
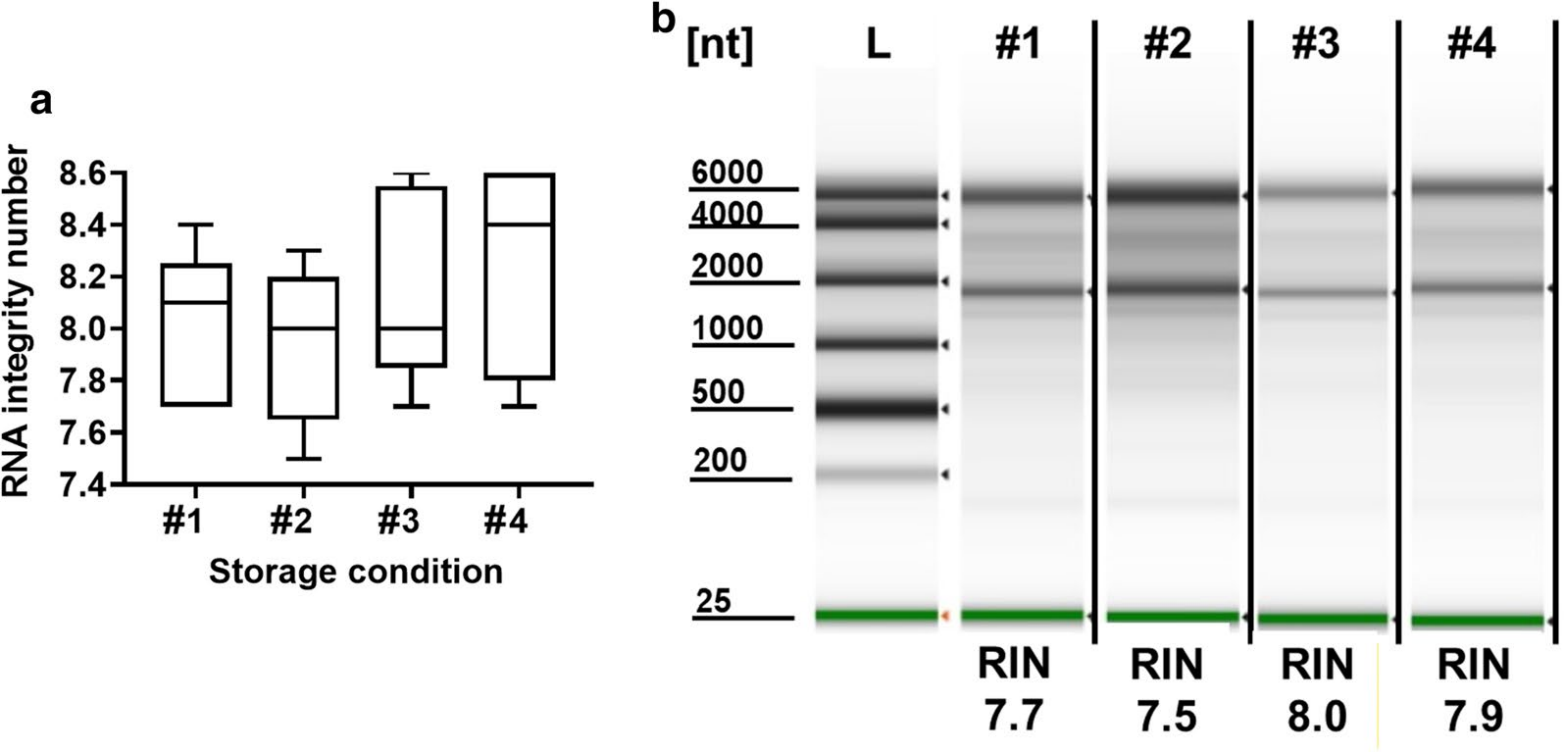
Long term storage and freeze–thaw cycles do not significantly impact RNA isolated from PAXgene tubes. The following conditions with corresponding numbers are represented in this figure: #1—baseline RNA that was immediately isolated; #2—isolated RNA stored at − 80 °C for one year; #3—isolated RNA exposed to five freeze–thaw cycles; #4—isolated RNA exposed to ten freeze–thaw cycles. **a** mean RIN values with SD for n = 5 healthy controls for each condition. **b** electropherogram and RIN values of a representative subject’s total RNA samples for each condition, *L* RNA ladder, *nt* nucleotide length. RIN analysis and electropherogram visualization were performed on the same gel and on the same day for all treatment conditions

We next investigated the effects of long-term storage on RNA integrity and the impact of multiple freeze–thaw cycles on total RNA by analyzing RNA integrity number (RIN) values as depicted in Additional file 7: Fig.S5. These values are calculated comparing 18S and 28S rRNA ratios where values greater than or equal to 7 are generally considered acceptable [29]. RNA samples isolated at baseline and stored for 1 year at − 80 °C exhibited no significant difference in overall RNA integrity determined by RIN (Fig. 2). Similarly, RNA samples subjected to five or ten freeze–thaw cycles did not show any significant degradation when analyzed by RIN values. Digitally captured electropherograms of total RNA samples used for RIN analysis are shown in Additional file 8: Fig.S6.

**Fig. 3:**
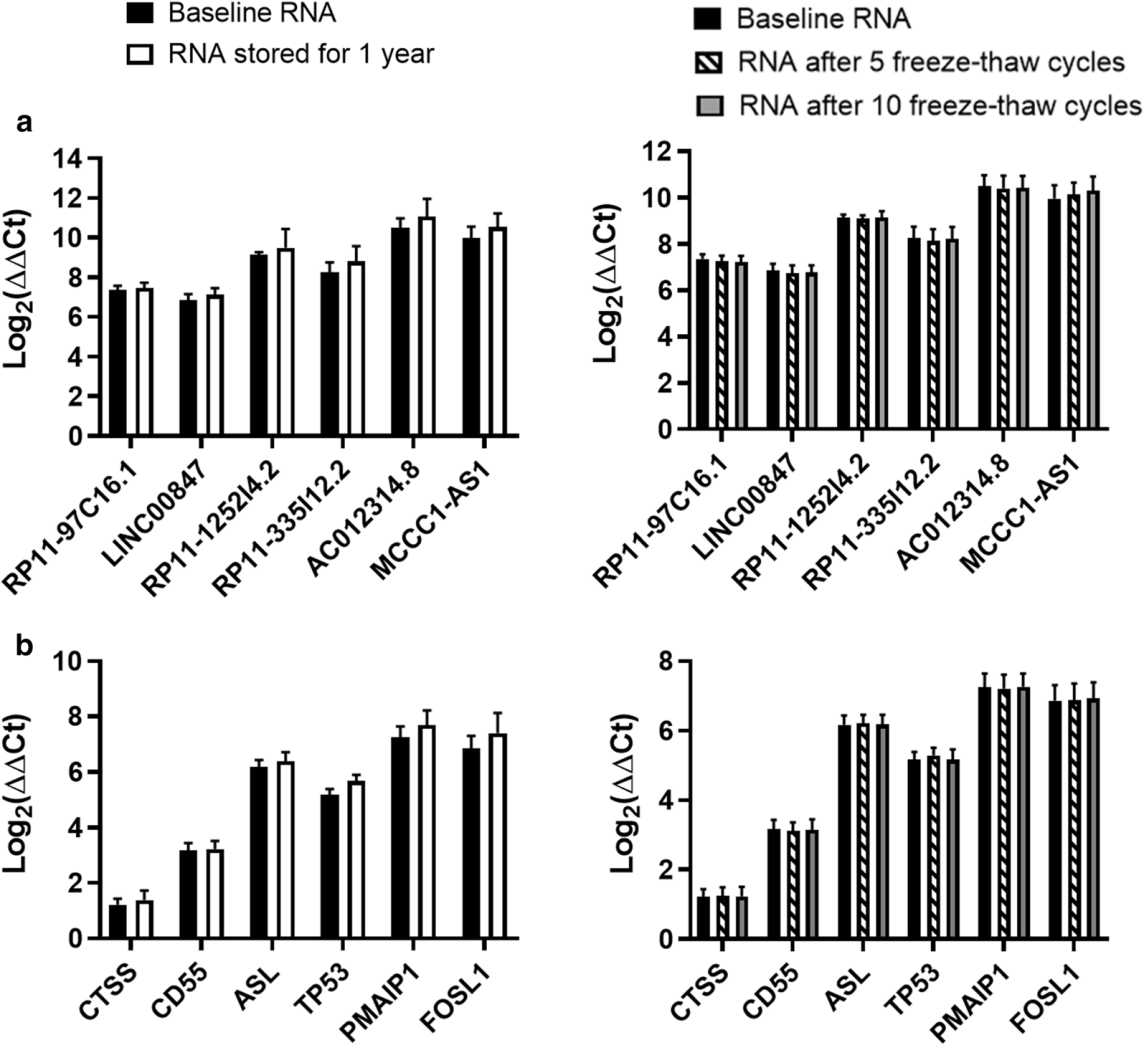
Long term storage and freeze–thaw cycles do not alter lncRNA or mRNA expression. qRT-PCR gene expression data were produced for **a** six lncRNAs and **b** six mRNAs. Baseline RNA immediately isolated from PAXgene Blood RNA tubes was compared to three treatment conditions including RNA storage at − 80 °C for one year, total RNA from PAXgene tubes exposed to five freeze–thaw cycles, and total RNA that was exposed to ten freeze–thaw cycles. Paired sample t-test was used to determine statistical significance. Bars represent mean Log_2_(ΔΔCt) with SD for n = 5 individual healthy subject’s baseline total RNA samples and after one of the three treatment conditions

To further assess the quality of each RNA template, cDNA was synthesized from each of the RNA samples at baseline, after storage for 1 year or after a series of five or ten freeze–thaw cycles. cDNA synthesized from each of these RNA templates did not exhibit significant differences in lncRNA expression values (Fig. 3a). As an additional comparison, canonical protein-coding genes were also measured to test whether − 80 °C storage or up to 10 freeze–thaw cycles would significantly alter expression of these mRNA targets as well (Fig. 3b). These mRNAs were selected from the same study comparing healthy control subjects and patients with relapsing–remitting multiple sclerosis [16, 18]. Just as we observed in lncRNAs, no significant differences in mRNA expression were found. Mean cycle threshold (Ct) values ± SD were compared across each experimental group. Amplification of all gene targets resulted in gene targets with Ct values that were less than 29. No significant difference in Ct value was observed across each of the experimental comparisons (Additional file 9: Fig.S7). Finally, similar results were also observed when cDNA samples were exposed to five or ten freeze–thaw cycles (Additional file 10: Fig.S8).

## Discussion

Since the beginning of this century, the number of documented RNA classes, and variety of noncoding transcripts has grown significantly. In the mid-2000s, the FANTOM3 project identified ~ 35,000 non-coding transcripts from ~ 10,000 distinct loci [30]. Recently a comprehensive analysis of lncRNAs from existing databases, published literature and novel RNA assemblies revealed that there are 270,044 lncRNA transcripts in the human genome [31]. PAXgene tubes are widely used in biobanking initiatives, clinical research studies, and molecular diagnostics to stabilize RNA species. Since PAXgene tubes are used with increasing frequency to correlate disease outcomes with RNA transcript levels, careful consideration should be given to downstream diagnostic applications to ensure reproducibility of RNA measurements over time.

We find that PAXgene tubes maintain the stability of lncRNA expression profiles when stored at − 80 °C for up to 1 year. We also find that storing total RNA or cDNA synthesized from fresh total RNA template or total RNA stored at − 80 °C for 1 year does not result in significant changes in lncRNA expression. While some month to month variation between datapoints is observed, we believe that these changes are inherent in the technical processing of samples and can disproportionately affect qRT-PCR targets of lower abundance. Additionally, while not directly examined in our work, previous studies have shown that expression measurements using shorter target amplicon lengths (70-250 bp) are less susceptible to variations in RNA integrity than larger PCR amplification products [29]. With the exception of *MCCC1-AS1* in our analysis, the monthly fluctuations observed among other lncRNAs resulted in overlapping 95% confidence intervals. Future studies seeking to develop novel assays based on mRNA or lncRNA expression values should examine expression differences over time, particularly for transcripts with lower overall expression values, to ensure that any technical variation or intrinsic stability does not adversely impact the accuracy, reproducibility or interpretability of assay results.

## Limitations

While the characteristics of each respective target should be assessed individually, one limitation of this study was the relatively small number of lncRNA and mRNA targets evaluated. Three laboratory technicians performed PAXgene RNA isolations, synthesized cDNA, and performed gene expression studies. This was intentional to simulate multiple operators that could potentially process PAXgene tubes in a clinical setting. PCR primers used in this study were printed onto 384-well plates robotically. Additional liquid handling steps could be implemented to further minimize operator to operator technical variation.

lncRNA and mRNA expression was normalized to *GAPDH* throughout our investigation. While *GAPDH* is widely accepted as an appropriate housekeeping gene, depending upon the use case, incorporation of other housekeeping genes could further reduce operator to operator or monthly expression variability. While single RIN assessments were performed for each of the experimental conditions in Fig. 2, these measurements were not performed for each sample across all experimental conditions in the study. Thus, we are unable to determine whether slight variations in RIN values could account for expression variability.

## Supplementary information

**Additional file 1:** **Table S1. **Genomic locations for qRT-PCR targets used in the study.

**Additional file 2:** **Figure S1. **RNA sequencing data for annotated lncRNAs differentially expressed among healthy controls and MS patients.

**Additional file 3:** **Figure S2. **Schematic representation of the lncRNA stability study**.**

**Additional file 4:** **Figure S3. **Monthly lncRNA expression analysis over the course of 1 year.

**Additional file 5:** **Table S2. **Summary of quality metrics across all total RNA samples isolated from PAXgene Blood RNA tubes.

**Additional file 6:** **Figure S4. **Summary of total RNA yield from PAXgene Blood RNA tubes processed during the 1 year study.

**Additional file 7:** **Figure S5. **Schematic representation of freeze-thaw experiments.

**Additional file 8:** **Figure S6. **Digitally captured full size electropherogram of total RNA samples processed for RNA Integrity Number analysis.

**Additional file 9:** **Figure S7. **Summary of lncRNA and mRNA expression values and representative amplification plots for mRNAs and lncRNAs.

**Additional file 10:** **Figure S8. **Long term cDNA storage and freeze-thaw cycles do not alter lncRNA or mRNA expression.

## Data Availability

The datasets used and/or analyzed during the current study are available from the corresponding author upon reasonable request. Previous RNA-sequencing data used to define PCR targets are publicly available [18].

## Abbreviations

CI: Confidence interval
CV: Coefficient of variation
FC: Fold change
FPKM: Fragments per kilobase of transcript per million mapped reads
GAPDH: Glyceraldehyde 3-phosphate dehydrogenase
lncRNA: Long non-coding RNA
mRNA: Messenger RNA
MS: Multiple sclerosis
qRT-PCR: Quantitative real-time polymerase chain reaction
RIN: RNA integrity number
SD: Standard deviation
SEM: Standard error of the mean
