# Supplementary material for: Longitudinal assessment and stability of long non-coding RNA gene expression profiles measured in human peripheral whole blood collected into PAXgene blood RNA tubes
Source: BMC Res Notes. 2020 Nov 12;13:531. doi: 10.1186/s13104-020-05360-3 (PMC7664084; doi:10.1186/s13104-020-05360-3)
Supplement: Supplementary file 1 — Additional file 1: Table S1. Genomic locations for qRT-PCR targets used in the study. [file 13104_2020_5360_MOESM1_ESM.pdf]

**Additional File 1, Table S1. Genomic locations for qRT-PCR targets used in the study**

| Gene ID | Class | Genomic location | Sense primer | Antisense primer |
|---|---|---|---|---|
| *RP11-97C16.1* | lncRNA | chr3:3152952-3153085 | TTTTGCAGAAAGCAGTTTCC | TTGCCATATAGGGCCAAAGT |
| *LINC00847* | lncRNA | chr5:180835290-180835417 | TCTCATGCCCTTGTACCACA | GAAACAAGGAGGCACATGGT |
| *RP11-1252I4.2* | lncRNA | chr5:177950350-177950456 | TTCATAACCACGCTGTTTGG | TCCTCAGGACAGAGCAGTGA |
| *RP11-335I12.2* | lncRNA | chr12:66257285-66257386 | TGAGGCTTGATGACCACAGA | TTCGACTTCACCCTTTGGTT |
| *AC012314.8* | lncRNA | chr19:54120234-54120339 | CTCTGCCTCCTCAACCAGAC | CTCCAGAGGGTTATGGGACA |
| *MCCC1-AS1* | lncRNA | chr3:183017444-183017573 | TCATGTTGCAAACCATGAAT | CACAACCACATGGGCTCTAC |
| *CTSS* | mRNA | chr1:150747785-150747880 | GGTGTCTACTATGAACCATCCTG | CACAAGCCAGTATTCTTTCCCA |
| *CD55* | mRNA | chr1:207336713-207336817 | GTCCCACCAACAGTTCAGAA | TTGAGCATTTGGTGTGGTGG |
| *ASL* | mRNA | chr7:66081801-66081918 | AGAGTGGGAAGCTTTGGGG | TTGCTGCCTTGAACATCCAC |
| *TP53* | mRNA | chr17:7674876-7674966 | GCCCCTCCTCAGCATCTTAT | GCACCACCACACTATGTCGA |
| *PMAIP1* | mRNA | chr18:59902649-59902751 | CTGGAAGTCGAGTGTGCTAC | AGGTTCCTGAGCAGAAGAGT |
| *FOSL1* | mRNA | chr11:65892941-65893039 | GACCACACCCTCCCTAACTC | CTGCTGCTACTCTTGCGATG |
| *GAPDH* | mRNA | chr12:6537318-6537618 | CTGCACCACCAACTGCTTAG | GTCTTCTGGGTGGCAGTGAT |
