# Supplementary material for: Longitudinal assessment and stability of long non-coding RNA gene expression profiles measured in human peripheral whole blood collected into PAXgene blood RNA tubes
Source: BMC Res Notes. 2020 Nov 12;13:531. doi: 10.1186/s13104-020-05360-3 (PMC7664084; doi:10.1186/s13104-020-05360-3)
Supplement: Supplementary file 2 — Additional file 2: Figure S1. RNA sequencing data for annotated lncRNAs differentially expressed among healthy controls and MS patients. [file 13104_2020_5360_MOESM2_ESM.pdf]

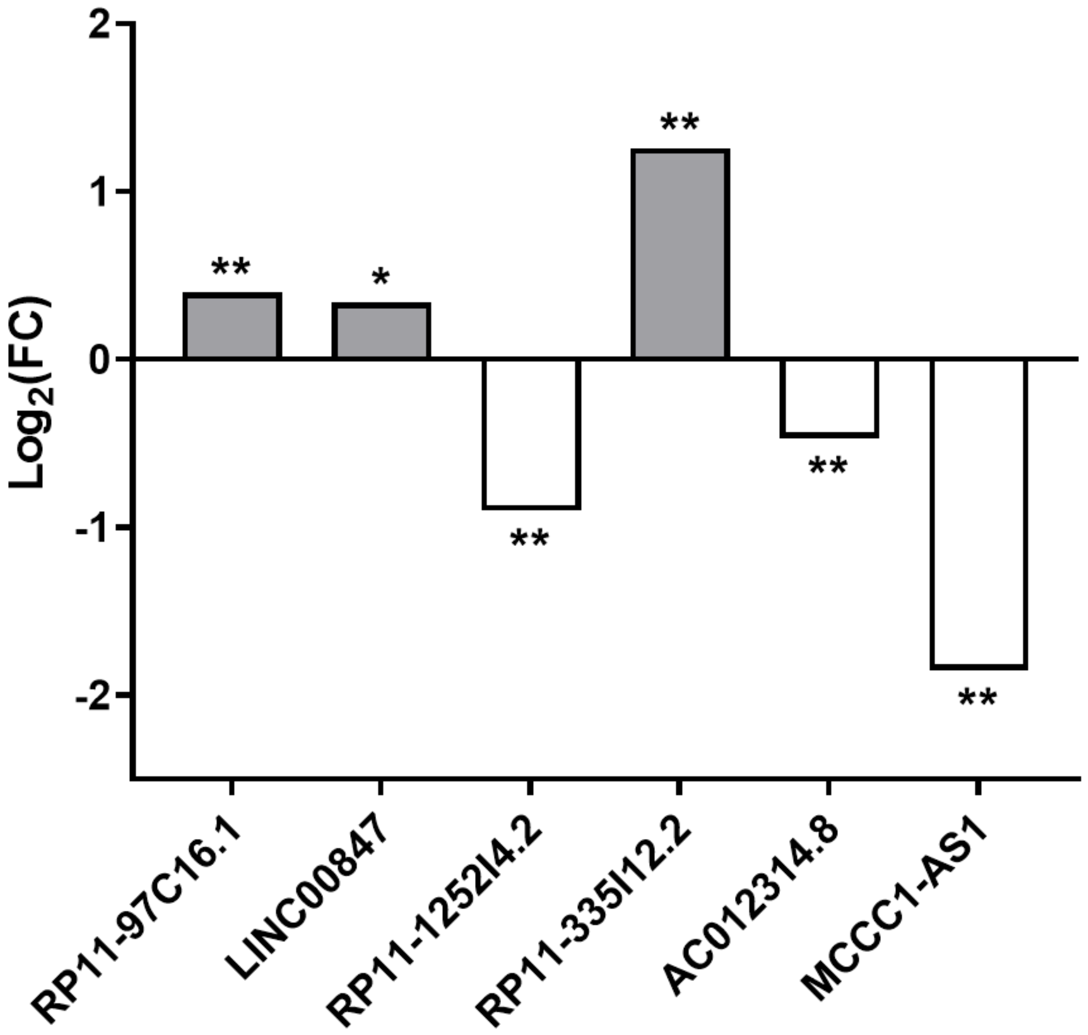

Additional File 2, Fig.S1. RNA sequencing data for annotated lncRNAs differentially expressed among healthy controls and MS patients. Log_2 fold change (FC) was calculated to visualize lncRNA overexpression (shaded bars) and under expression (clear bars) in relapsing-remitting multiple sclerosis patients compared to healthy controls. Healthy control n=8; MS group n=6. p-values are reported using Student's t-test with Welch's correction, * $p<0.05$, ** $p<0.01$. RNA sequencing values were determined in previous studies [18] and calculated as fragments per kilobase per million mapped reads (FPKM).
