# Supplementary material for: Longitudinal assessment and stability of long non-coding RNA gene expression profiles measured in human peripheral whole blood collected into PAXgene blood RNA tubes
Source: BMC Res Notes. 2020 Nov 12;13:531. doi: 10.1186/s13104-020-05360-3 (PMC7664084; doi:10.1186/s13104-020-05360-3)
Supplement: Supplementary file 3 — Additional file 3: Figure S2. Schematic representation of the lncRNA stability study. [file 13104_2020_5360_MOESM3_ESM.pdf]

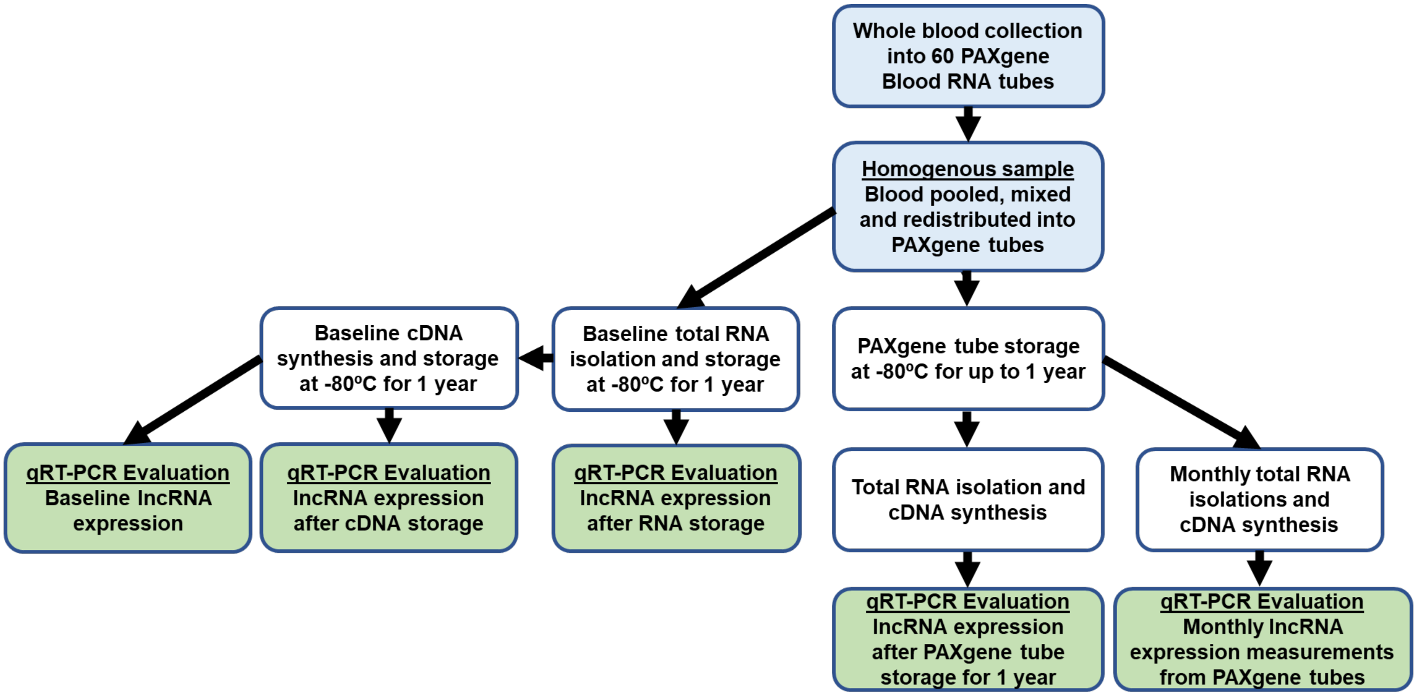

Additional File 3. Fig.S2. Schematic representation of the lncRNA stability study. Blood from healthy subjects (n=5) was pooled to make a homogenous mixture and aliquoted into individual PAXgene Blood RNA tubes. Total RNA was immediately isolated from five PAXgene tubes followed by cDNA synthesis and qRT-PCR to evaluate baseline lncRNA expression values. The remaining 11 sets of five PAXgene tubes were stored at -80ºC for up to one year and used for total RNA isolation, cDNA synthesis and qRT-PCR expression measurements at each of the remaining monthly time points.
