# Supplementary material for: Longitudinal assessment and stability of long non-coding RNA gene expression profiles measured in human peripheral whole blood collected into PAXgene blood RNA tubes
Source: BMC Res Notes. 2020 Nov 12;13:531. doi: 10.1186/s13104-020-05360-3 (PMC7664084; doi:10.1186/s13104-020-05360-3)
Supplement: Supplementary file 4 — Additional file 4: Figure S3. Monthly lncRNA expression analysis over the course of 1 year. [file 13104_2020_5360_MOESM4_ESM.pdf]

Wylezinski et al.
Additional File 4, Figure S3

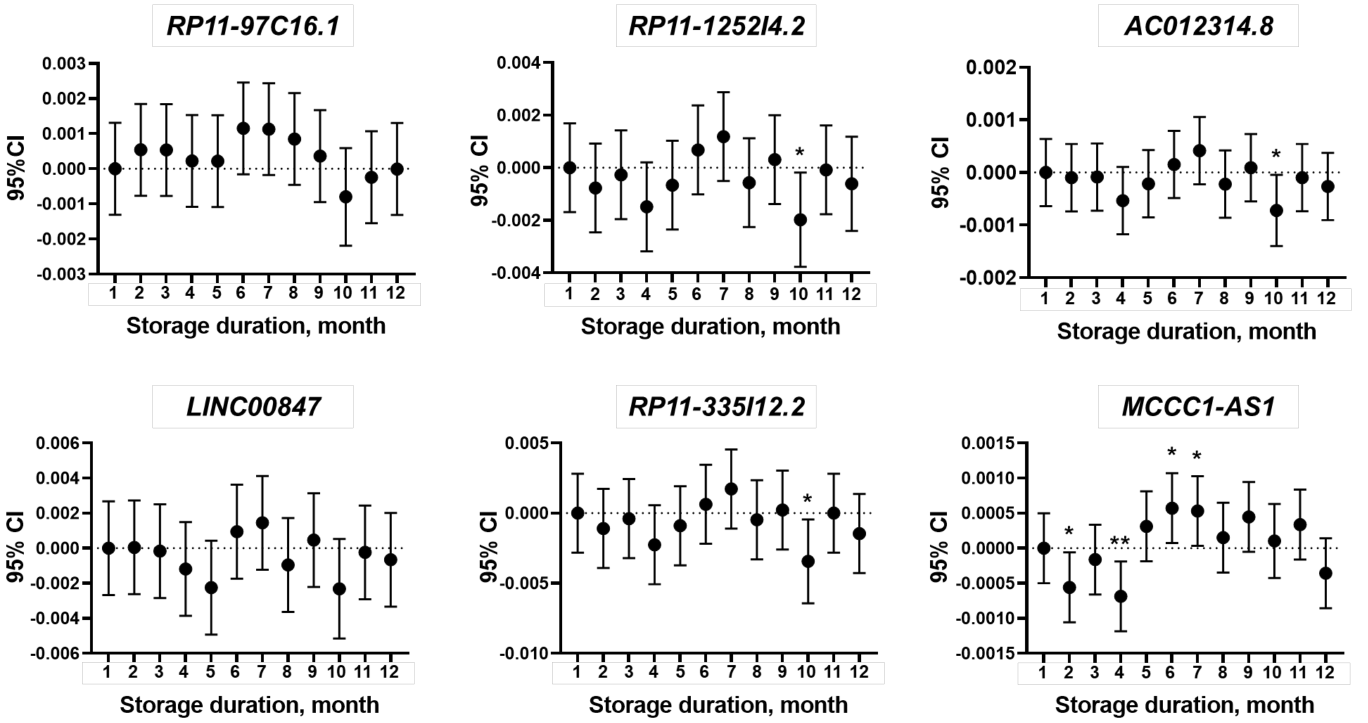

Additional File 4, Fig.S3. Monthly lncRNA expression analysis over the course of one year. Blood from n=5 healthy controls was pooled to make a homogenous mixture and aliquoted into individual PAXgene Blood RNA tubes for RNA isolation at each timepoint. 95% confidence intervals (CI) were calculated for the differences between normalized Ct values (ΔΔCts) of baseline RNA samples (total RNA isolated immediately after blood collection) and total RNA samples isolated consecutively each month after initiation of the one-year study. Samples were stored in PAXgene tubes at -80ºC for one year. Statistical significance was assessed by one-way ANOVA analysis with Sidak's multiple comparisons, * $p<0.05$, ** $p<0.01$, n=5 PAXgene tubes at each timepoint.
