# Supplementary material for: Longitudinal assessment and stability of long non-coding RNA gene expression profiles measured in human peripheral whole blood collected into PAXgene blood RNA tubes
Source: BMC Res Notes. 2020 Nov 12;13:531. doi: 10.1186/s13104-020-05360-3 (PMC7664084; doi:10.1186/s13104-020-05360-3)
Supplement: Supplementary file 5 — Additional file 5: Table S2. Summary of quality metrics across all total RNA samples isolated from PAXgene Blood RNA tubes. [file 13104_2020_5360_MOESM5_ESM.pdf]

Additional File 5, Table S2. Summary of quality metrics across all total RNA samples isolated from PAXgene Blood RNA tubes

Parameters for RNA samples (n = 60)	Concentration, ng/µL	A260/A280	A260/A230
Mean	330.62	2.06	2.04
SD	73.58	0.01	0.27
Standard error of the mean	±9.50	±0.002	±0.03
Coefficient of variation (%)	22.26	0.72	13.21
