# Supplementary material for: Longitudinal assessment and stability of long non-coding RNA gene expression profiles measured in human peripheral whole blood collected into PAXgene blood RNA tubes
Source: BMC Res Notes. 2020 Nov 12;13:531. doi: 10.1186/s13104-020-05360-3 (PMC7664084; doi:10.1186/s13104-020-05360-3)
Supplement: Supplementary file 6 — Additional file 6: Figure S4. Summary of total RNA yield from PAXgene Blood RNA tubes processed during the 1 year study. [file 13104_2020_5360_MOESM6_ESM.pdf]

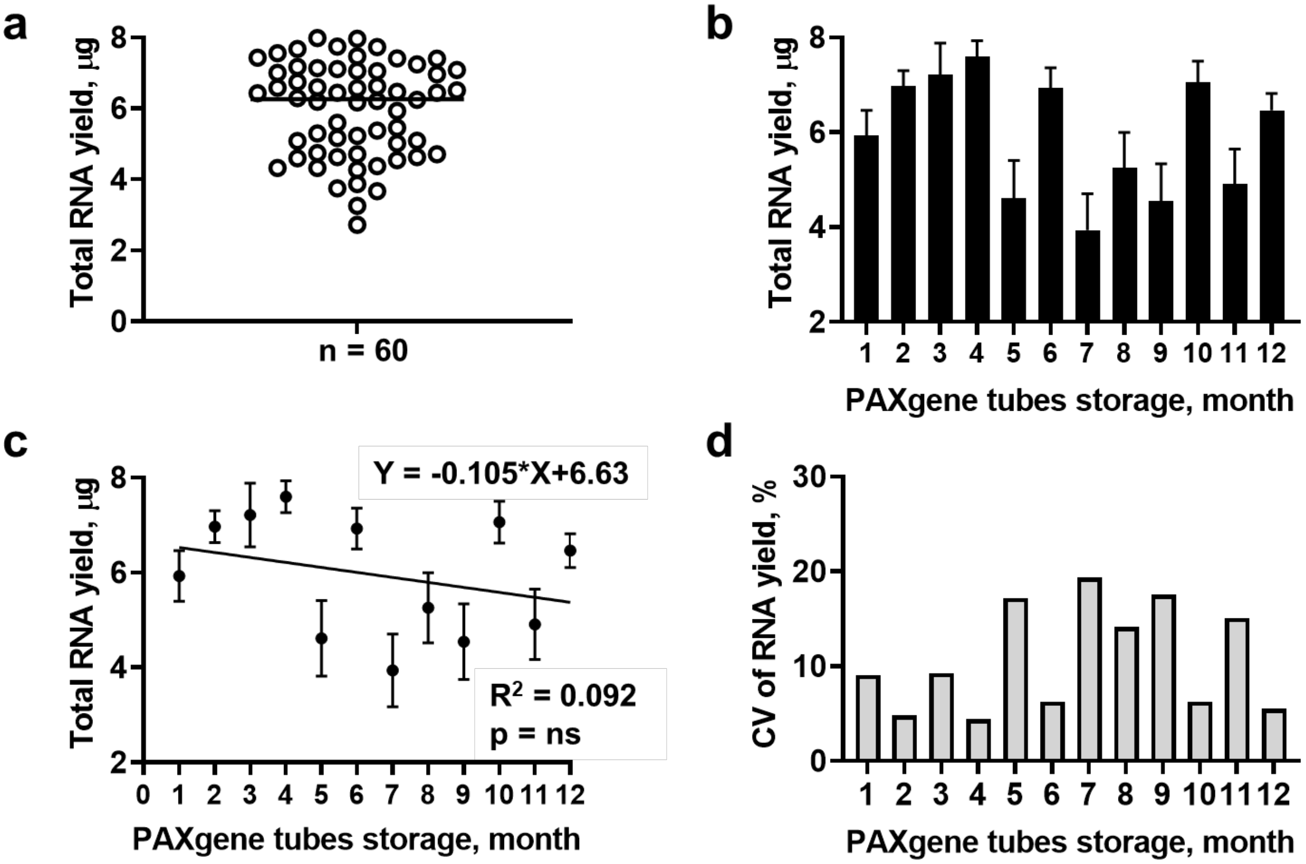

Additional File 6, Fig.S4. Summary of total RNA yield from PAXgene Blood RNA tubes processed during the one-year study. Peripheral whole blood from n=5 healthy controls was pooled and aliquoted into individual PAXgene Blood RNA tubes for RNA isolation at each timepoint. **a** - total RNA yield was measured for each processed PAXgene tube. **b** - mean total RNA yield with SD for each monthly RNA isolation. **c** - linear regression analysis of total RNA yield obtained at each timepoint. **d** - coefficient of variation (CV) of RNA yield for each RNA isolation; n=5 PAXgene tubes per timepoint.
