# Supplementary material for: Longitudinal assessment and stability of long non-coding RNA gene expression profiles measured in human peripheral whole blood collected into PAXgene blood RNA tubes
Source: BMC Res Notes. 2020 Nov 12;13:531. doi: 10.1186/s13104-020-05360-3 (PMC7664084; doi:10.1186/s13104-020-05360-3)
Supplement: Supplementary file 7 — Additional file 7: Figure S5. Schematic representation of freeze-thaw experiments. [file 13104_2020_5360_MOESM7_ESM.pdf]

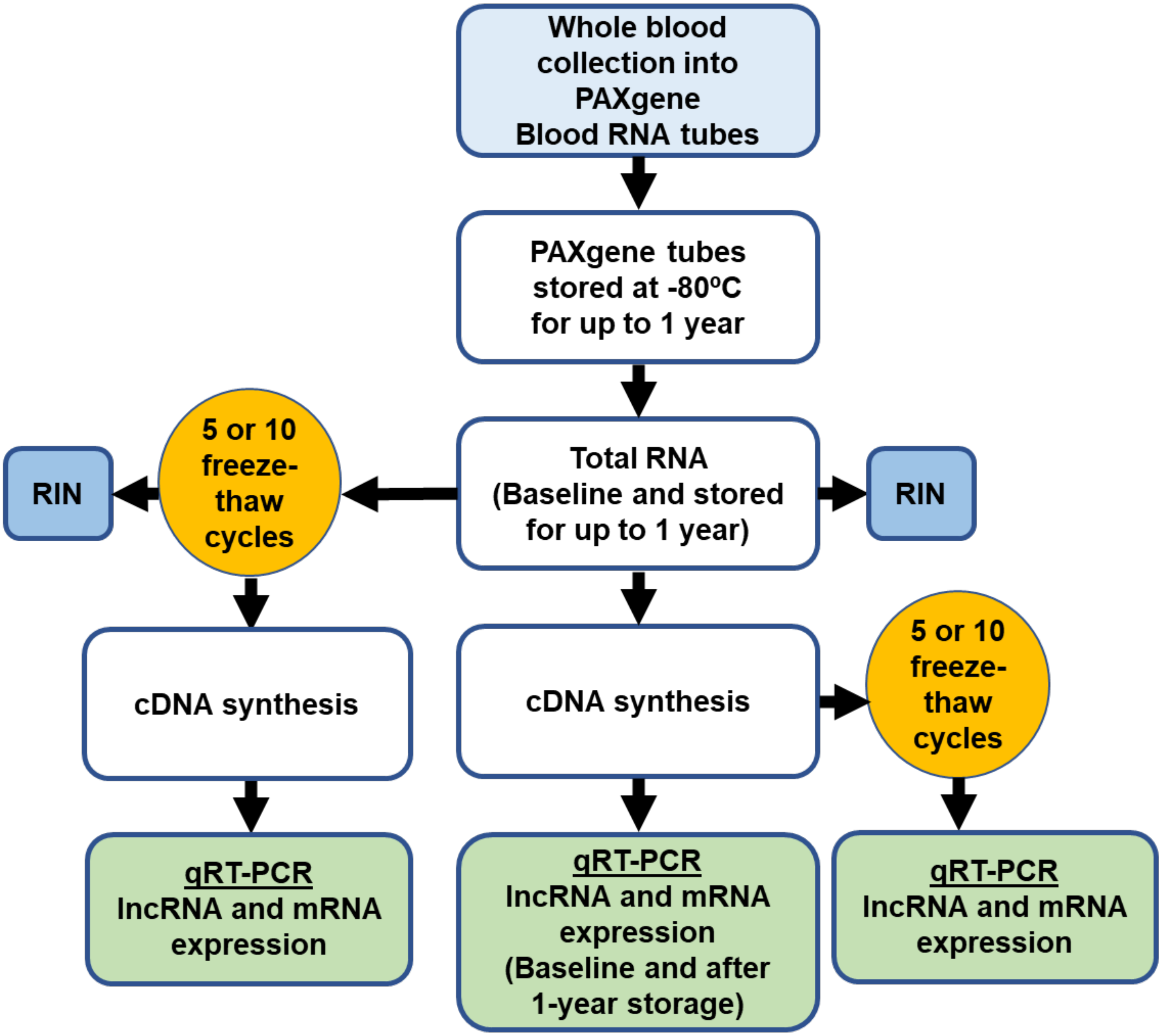

Additional File 7. Fig.S5. Schematic representation of freeze-thaw experiments. Whole blood was collected from n=5 healthy subjects into PAXgene Blood RNA tubes. qRT-PCR studies of lncRNA and mRNA expression were performed at the time of isolation and after one year of storage. Total RNA samples isolated from PAXgene tubes or freshly synthesized cDNA samples were exposed to five or ten freeze-thaw cycles followed by lncRNA and mRNA expression analysis. Quality of all RNA samples was assessed using RNA Integrity Number (RIN) measurements.
