# Supplementary material for: Longitudinal assessment and stability of long non-coding RNA gene expression profiles measured in human peripheral whole blood collected into PAXgene blood RNA tubes
Source: BMC Res Notes. 2020 Nov 12;13:531. doi: 10.1186/s13104-020-05360-3 (PMC7664084; doi:10.1186/s13104-020-05360-3)
Supplement: Supplementary file 8 — Additional file 8: Figure S6. Digitally captured full size electropherogram of total RNA samples processed for RNA Integrity Number analysis. [file 13104_2020_5360_MOESM8_ESM.pdf]

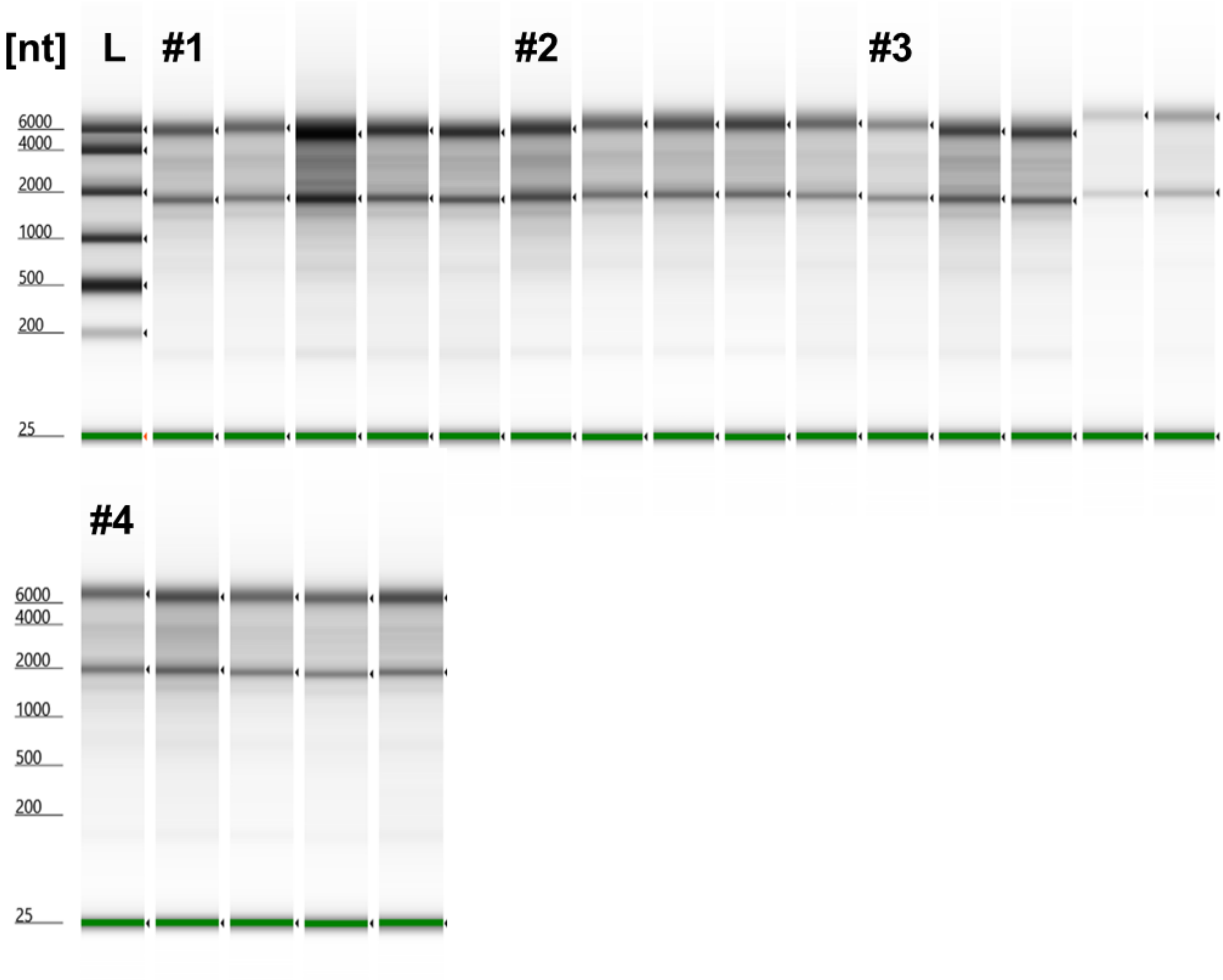

Additional File 8, Fig.S6. Digitally captured full size electropherogram of total RNA samples processed for RNA Integrity Number analysis. This is the original image that includes all samples examined during a single RIN analysis on the same day. Lanes labeled #1-4 correspond to the cropped images presented in Figure 2b. Conditions are as follows: #1 - baseline RNA that was immediately isolated with no storage; #2 - RNA isolated and stored at -80°C for one year; #3 - isolated RNA exposed to five freeze-thaw cycles; #4 - isolated RNA exposed to ten freeze-thaw cycles. L – RNA ladder, nt – nucleotide length.
