# Supplementary material for: Longitudinal assessment and stability of long non-coding RNA gene expression profiles measured in human peripheral whole blood collected into PAXgene blood RNA tubes
Source: BMC Res Notes. 2020 Nov 12;13:531. doi: 10.1186/s13104-020-05360-3 (PMC7664084; doi:10.1186/s13104-020-05360-3)
Supplement: Supplementary file 9 — Additional file 9: Figure S7. Summary of lncRNA and mRNA expression values and representative amplification plots for mRNAs and lncRNAs. [file 13104_2020_5360_MOESM9_ESM.pdf]

a

Target gene ID	Class	Baseline RNA		RNA stored for 1 year		5 freeze-thaw cycles		10 freeze-thaw cycles	
		Mean Ct	SD	Mean Ct	SD	Mean Ct	SD	Mean Ct	SD
CTSS	mRNA	19.74	0.16	20.06	0.29	19.91	0.18	19.75	0.24
CD55	mRNA	21.67	0.36	21.90	0.44	21.79	0.41	21.66	0.44
TP53	mRNA	23.69	0.13	24.35	0.25	23.96	0.34	23.68	0.33
ASL	mRNA	24.69	0.19	25.06	0.28	24.89	0.23	24.68	0.27
PMAIP1	mRNA	25.76	0.31	26.37	0.45	25.88	0.31	25.78	0.33
FOSL1	mRNA	25.38	0.36	26.08	0.60	25.55	0.40	25.45	0.41
LINC00847	lncRNA	25.38	0.21	25.81	0.27	25.42	0.23	25.28	0.25
RP11-1252I4.2	lncRNA	27.64	0.21	28.18	0.96	27.75	0.31	27.67	0.33
AC012314.8	lncRNA	28.99	0.39	29.71	0.73	29.05	0.46	28.94	0.47
RP11-97C16.1	lncRNA	25.88	0.14	26.13	0.37	25.92	0.22	25.75	0.29
RP11-335I12.2	lncRNA	26.77	0.40	27.49	0.64	26.82	0.44	26.75	0.48
MCCC1-AS1	lncRNA	28.46	0.45	29.21	0.55	28.81	0.49	28.80	0.55
GAPDH	mRNA	18.51	0.10	18.68	0.24	18.67	0.20	18.52	0.14

mRNAs

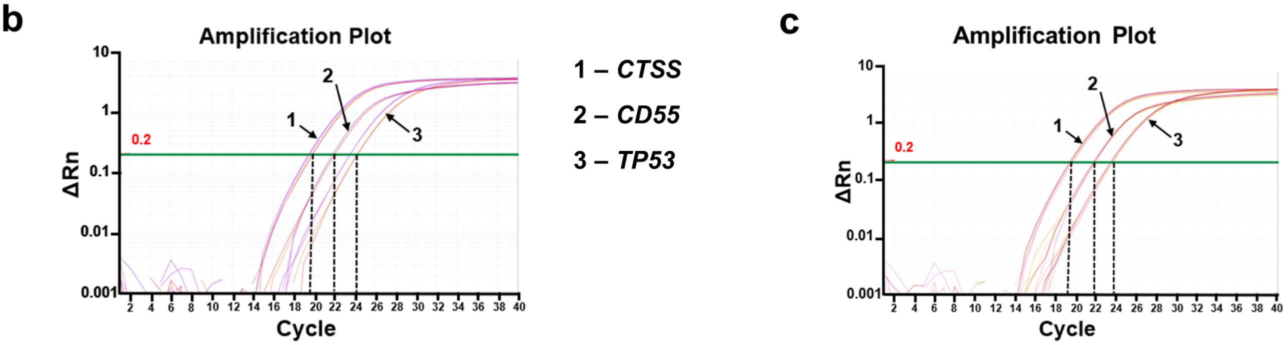

lncRNAs

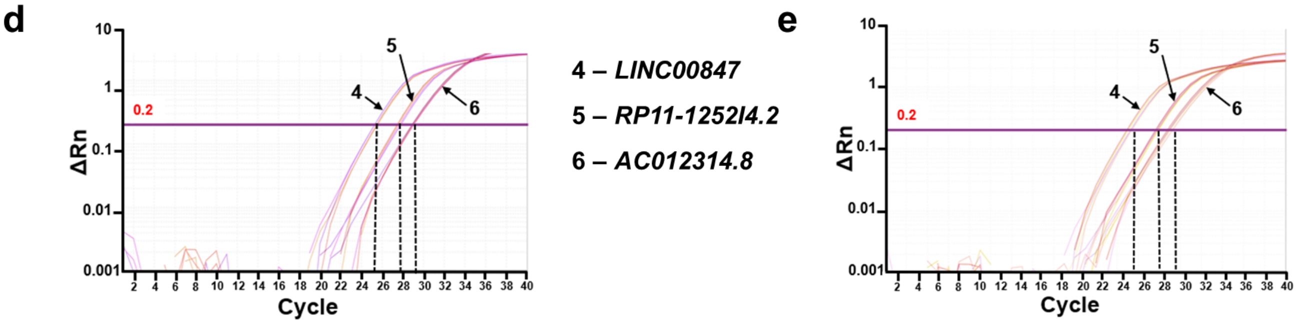

Additional File 9, Fig.S7. Summary of lncRNA and mRNA expression values and representative amplification plots for mRNAs and lncRNAs. a - summary of mean Ct values with SD from qRT-PCR data collected from RNA samples exposed to various storage conditions. **b, c** - amplification plot for *CTSS, CD55,* and *TP53* mRNAs. **d, e** - amplification plot for *LINC00847, RP11-1252I4.2* and *AC012314.8* lncRNAs. Each plot shows amplification curves from baseline RNA that was never exposed to storage conditions. Panels **b** and **d** include amplification curves from total RNA samples that were stored at -80°C for one year. Panels **c** and **e** include amplification curves generated from RNA after five freeze-thaw cycles and after ten freeze-thaw cycles. Amplification plots were generated using QuantStudio12K Flex software. Vertical black dashed lines on the plots show the mean threshold cycle (Ct) for each target gene at the intersection between an amplification curve and the set threshold (ΔRn=0.2).
