# Supplementary material for: Longitudinal assessment and stability of long non-coding RNA gene expression profiles measured in human peripheral whole blood collected into PAXgene blood RNA tubes
Source: BMC Res Notes. 2020 Nov 12;13:531. doi: 10.1186/s13104-020-05360-3 (PMC7664084; doi:10.1186/s13104-020-05360-3)
Supplement: Supplementary file 10 — Additional file 10: Figure S8. Long term cDNA storage and freeze-thaw cycles do not alter lncRNA or mRNA expression. [file 13104_2020_5360_MOESM10_ESM.pdf]

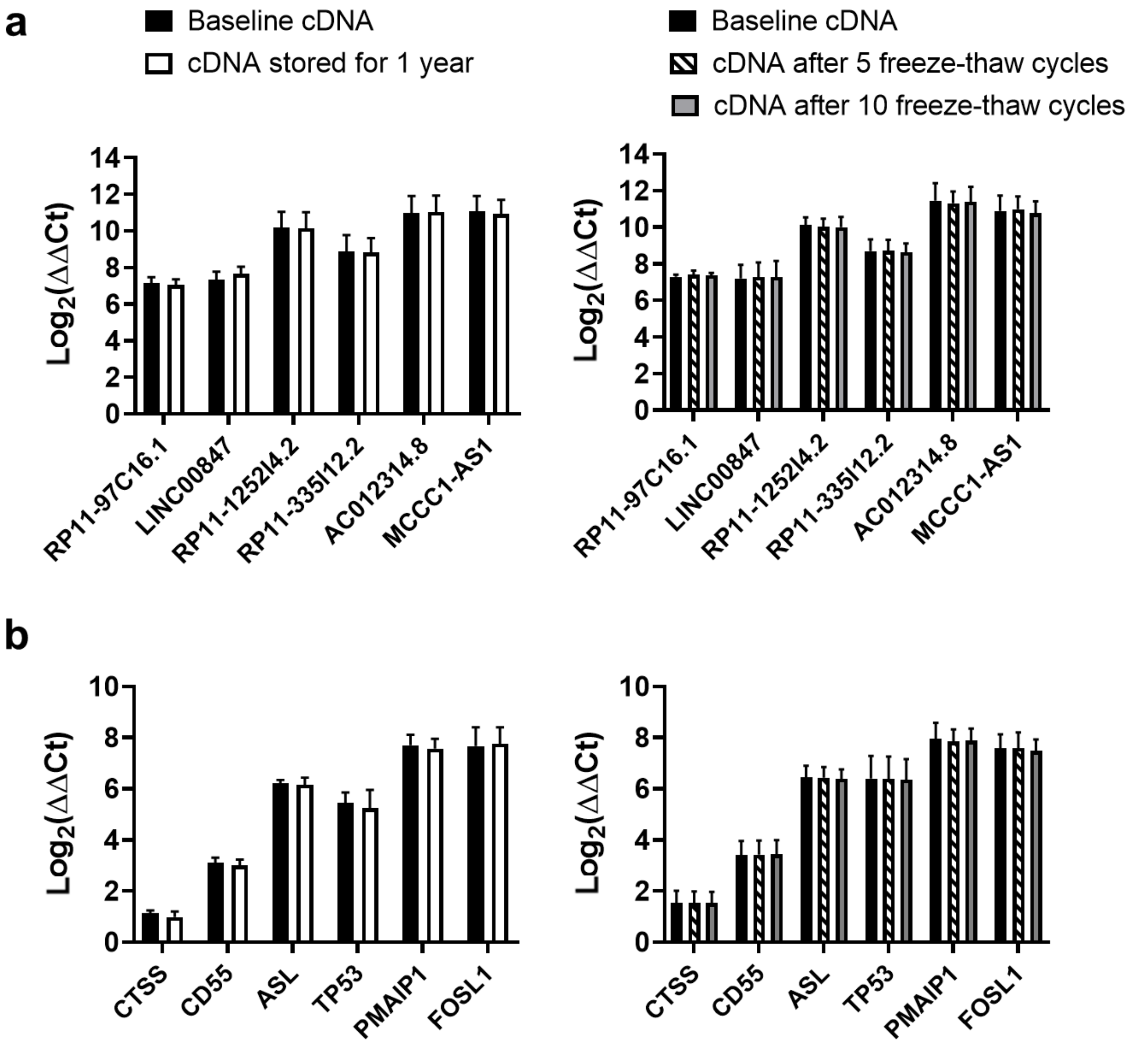

Additional File 10, Fig.S8. Long term cDNA storage and freeze-thaw cycles do not alter lncRNA or mRNA expression. qRT-PCR gene expression data were produced for **a** - six lncRNAs and **b** - six mRNAs. Baseline cDNA was synthesized using RNA freshly isolated from PAXgene Blood RNA tubes and was compared to three treatment conditions including cDNA storage at -80°C for one year, cDNA exposed to five freeze-thaw cycles, and cDNA that was exposed to ten freeze-thaw cycles. Paired sample t-test was used to determine statistical significance. Bars represent mean $Log_2(\Delta\Delta Ct)$ with SD for n=5 individual healthy subject's baseline cDNA samples and after one of the three treatment conditions.
